# Cadaveric study of ergonomics and performance using a robotic exoscope with a head-mounted display in spine surgery

**DOI:** 10.1007/s11701-023-01777-7

**Published:** 2024-01-10

**Authors:** Matthias Demetz, Anto Abramovic, Aleksandrs Krigers, Marlies Bauer, Sara Lener, Daniel Pinggera, Johannes Kerschbaumer, Sebastian Hartmann, Helga Fritsch, Claudius Thomé, Christian F. Freyschlag

**Affiliations:** 1 Department of Neurosurgery, Medical University of Innsbruck, Anichstr. 35, 6020 Innsbruck, Austria; 2 Institute of Clinical and Functional Anatomy, Medical University of Innsbruck, Müllerstr. 59, 6020 Innsbruck, Austria

**Keywords:** RoboticScope, Exoscope, Microsurgery, Neurosurgery, Ergonomics

## Abstract

The conventional microscope has the disadvantage of a potentially unergonomic posture for the surgeon, which can affect performance. Monitor-based exoscopes could provide a more ergonomic posture, as already shown in pre-clinical studies. The aim of this study was to test the usability and comfort of a novel head-mounted display (HMD)-based exoscope on spinal surgical approaches in a simulated OR setting. A total of 21 neurosurgeons naïve to the device were participated in this prospective trial. After a standardized training session with the device, participants were asked to perform a single-level thoracolumbar decompression surgery on human cadavers using the exoscope. Subsequently, all participants completed a comfort and safety questionnaire. For the objective evaluation of the performance, all interventions were videotaped and analyzed. Twelve men and nine women with a mean age of 34 (range: 24–57) were participating in the study. Average time for decompression was 15 min (IqR 9.6; 24.2); three participants (14%) terminated the procedure prematurely. In these dropouts, a significantly higher incidence of back/neck pain (*p* = 0.002 for back, *p* = 0.046 for neck pain) as well as an increased frequency of HMD readjustments (*p* = 0.045) and decreased depth perception (*p* = 0.03) were documented. Overall, the surgeons’ satisfaction with the exoscope was 84% (IqR 75; 100). Using a standardized, pre-interventional training, it is possible for exoscope-naïve surgeons to perform sufficient spinal decompression using the HMD-based exoscope with a high satisfaction. However, inaccurate HMD setup prior to the start of the procedure may lead to discomfort and unsatisfactory results.

## Introduction

In contemporary neurosurgery, the use of an operating microscope (OM) is vital for the majority of procedures especially in minimally invasive approaches [1, 2]. Despite continuous technical advances, the conventional operating microscope struggles with disadvantages regarding the unergonomic posture for the surgeon based on the need for a connected optic axis from lens to eyepiece [3–5]. This disadvantage may lead to decreased surgical performance due to a higher incidence of work-related musculoskeletal disorders that has been shown in long-term studies for all microsurgeons compared to surgeons not using microscopes [5–9].

In comparison to conventional microscopes, exoscopes harbor the considerable advantage of projecting the images to external mobile screens, allowing the surgeon to be in a more favorable posture by subdivision of lens and visualization [8, 10, 11]. Despite these advantages, the depth of visible field and visual quality at higher magnification levels have been reported to pose limitations to surgeons performing microsurgical procedures because of the distance between surgeon and monitor [11–13].

The robot-controlled exoscope (Roboticscope, RS; BHS Technologies GmbH, Innsbruck, Austria) is able to project the images from two robot-controlled cameras on external virtual-reality-like displays (head-mounted display, HMD). The robotic arm is controlled hands-free by head gestures guided by a software interface. For safety reasons, the RS only moves according to head gestures when a foot pedal is pressed. This allows the surgeon to adopt a more comfortable posture without changing the camera position [14–16].

Following our initial evaluation of a customized microsurgical training tool in conjunction with the RS, a cadaver study was planned as a follow-up study to evaluate the applicability of the RS for spinal decompression in a pre-clinical setting and to further evaluate the possibility of a smooth transition of the RS to the operating room (OR) [17].

## Material and methods

### Participants

Twenty-one neurosurgeons, who had no previous experience with the HMD-based exoscope, were invited to participate in this pre-clinical prospective study. After an initial 30-min training session, including completion of a standardized ten-step microsurgical course, participants were asked to perform a routine microsurgical decompression of the thoracolumbar spine using the HMD-based exoscope.

### Operative procedure

For the surgical procedures, specimens provided by two human body donors of the Institute of Clinical Functional Anatomy Innsbruck were used.

All surgical interventions were performed with the help of surgical instruments identical to those used in clinical practice, including a high-speed drill. The surgical procedures included unmagnified skin incision with subsequent subcutaneous/muscular dissection and determination of the anatomical bony landmarks. Single-level exposure was created and a standard Caspar lumbar retractor system was inserted. Following this, setup and adjustment of the HMD-based exoscope took place. The exoscope was inserted as the conventional microscope would have been used to simulate a standard procedure. Participants were asked to perform lumbar decompression (interlaminar fenestration, flavectomy, identification of the dura, nerve root, and disc). After completion of these steps, the surgical procedure was considered complete.

### Video analysis

**Fig. 1:**
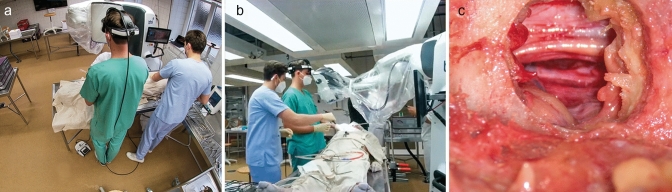
Different camera angles (top view, **a**; frontolateral view, **b**; camera view, **c**) were used to track and analyze the surgical steps as well as the need for technical assistance of the study participants

A total of three external cameras were used at different angles (top view, frontolateral view, microscopic view; Fig. 1) to capture the surgical procedure and the actions and position of the surgeons. The video was divided into four equal quarters (Start: positioning of the burr at the lamina; End: last command executed with the HMD-based exoscope) to show a possible learning curve with the handling of the exoscope. Two of the authors (MD, AA) performed a post-interventional analysis in terms of time required from laminotomy to final decompression, as well as the exoscope-related intraoperative error rate, requirement of technical assistance, and premature dropouts using a predefined eCRF.

### Postoperative survey

After performing the procedure, participants were asked to complete a questionnaire on surgical comfort and usability of the HMD-based exoscope. In addition to technical satisfaction (video quality [measured from 0 (poor) to 100% (excellent)]; overall satisfaction, mobility, comfort, and ease of use), ergonomics (headache, back-/neck pain [Visual Analog Scale, VAS]) and safety with the use of the device were questioned.

### Statistical analysis

Data were documented in pseudonymous form using the participant ID. Statistical analysis was performed using SPSS v 27.0 (SPSS, IBM Corporation, Armonk, New York, NY, USA). Interval-scaled data were sampled as a function of normal distribution using Student's *T* test or Mann–Whitney U test. Analysis of dichotomized data was performed with Chi^2^ or Fisher-Exact. In addition, Spearman correlation analysis was performed to identify possible associations between the microsurgical training course and thoracolumbar decompression outcomes. A *p* value < 0.05 was considered statistically significant.

## Results

### Demographics

Twenty-one neurosurgeons (twelve male, nine female) with a median age of 34 years (inter-quartile range [IQR] 31; 40) participated in this study. Median neurosurgical experience was 7 years (IQR 3; 12). 91% (19/21) of participants were right-handed, and more than half (11/21; 52%) suffered from any form of refractive error. Eight participants (39%) had experience with game consoles, ten participants (48%) had experience with computer games, and only one participant (5%) had previous experience with virtual reality.

The participants needed a median of 9.8 min (IQR 8.3; 12.3) to complete the pre-interventional standardized ten-step microsurgical training course. Median satisfaction with image quality was 78.5% (IQR 68.5; 88.8). The median satisfaction of participants in using the HMD-based exoscope during the training was 82%.

### Operative procedure

The median time to perform thoracolumbar decompression was 14.8 min (IQR 9.6; 24.2). The median number of commands performed for Q1–Q4 were 7, 5, 5, and 10, respectively, with command error rates of 14%, 20%, 0%, and 20%. Seven participants (33.3%) required technical assistance during the procedure, and eleven participants (52.4%) had to manually adjust the HMD to maintain sufficient vision. In ten participants (47.6%), the exoscope reached an angulation which allowed no further movement without performance of the automated rerouting of the robot arm. In six participants (28.6%), the exoscope had to be manually readjusted to provide a sufficient viewing angle.

Three participants (14.3%) terminated the surgical procedure prematurely. Compared to the other participants, these participants reported significantly lower overall satisfaction (50% vs. 87%, *p* = 0.045) as well as decreased depth perception (40% vs. 80%, *p* = 0.030); furthermore, higher frequency of manual HMD readjustments (*n* = 2 vs. *n* = 0, *p* = 0.39) and increased neck (VAS 3 vs. 2, *p* = 0.046) and/or back pain (VAS 3 vs. 0, *p* = 0.003) were shown during the procedure. In addition, participants who had to discontinue the procedure indicated that they would have needed more time during the microsurgical training course (*p* = 0.046).

### Post-interventional surveys

The overall satisfaction of the participants in using the HMD-based exoscope for thoracolumbar decompression was 84% (IQR 75, 100). Mobility, comfort, and ease of use were 72% (IQR 55, 80), 80% (IQR 60, 87), and 76% (IQR 65, 94), respectively. Median satisfaction with the image quality of the HMD-based exoscope was 80% (IQR 67, 91). The median satisfaction regarding the depth perception was 78% (IQR 58, 85). During performance of thoracolumbar decompression, participants reported median neck pain of Visual Analog Scale (VAS) 2 (IQR 2, 3). Back pain (defined as a VAS > 1) occurred in 19% of participants with a range of VAS 2–3.

### Comparison of microsurgical training course with thoracolumbar decompression

The time to complete the microsurgical training course correlated significantly with the time to perform thoracolumbar decompression (Spearman’s *ρ* = 0.829, *p* < 0.001). Participants who felt confident using the HMD-based exoscope after completing the ten-step microsurgical training course reported the use of the exoscope during thoracolumbar decompression felt easy (*ρ* = 0.82, *p* < 0.001). Participants who reported poor image quality during decompression were significantly more likely to report feeling “uncomfortable” using the exoscope during the pre-interventional test (*ρ* = 0.781, *p* < 0.001).

## Discussion

The results of this study were generated as the second part of a prospective study during an introductory workshop for the HMD-based exoscope [17]. Our study showed that after training with a standardized ten-step microsurgical exercise, there is a high level of confidence in the use of the RS for thoracolumbar decompressive surgery.

Prior studies have already evaluated the RS on cadavers as well as in the clinical setting for ENT procedures. They reported hands-free control and quality of visualization as major advantages [14]. A previous trial of our study group using a customized microsurgical training tool assessment has shown a high satisfaction with usability (80%) and quality of visualization (82%) among the participants as well as a steep learning curve during the ten-step microsurgical training. The post-interventional questionnaire showed that 88% of the participants reported to feel safe to use the RS in the OR with technical assistance [17].

Participants who had to discontinue surgery complained of dissatisfaction with the device, largely due to limited depth perception. Incorrect HMD setup at the beginning of surgery can compromise visual quality and lead to discomfort and premature termination of surgery due to back and neck pain. The fact that more than half of the participants still required technical assistance (e.g., HMD adjustment) indicates the potential of further improvements of the HMD. The accurate positioning of the HMD before the start of the procedure seems to be crucial for the comfort of the surgeons during surgery. The three participants with premature dropout showed a significantly higher frequency of HMD readjustments and significantly decreased depth perception, what might be caused by a minor displacement or suboptimal initial adjustment of the HMD. This could lead to physical discomfort and dizziness as described in our previous study, but also back and neck pain as shown in our results [17]. The weight of the HMD as well as the wire-based connection between the device and the HMD may have played a role leading to a higher necessity of technical assistance. Overall, some technical assistance was required by more than half of the participants, which might be because of the novelty with the device and high-quality standards the participants were used to.

Apart from the technical opportunities of a newly designed visualization device, one of the major concerns during the implementation of new imaging techniques is the uncertainty whether surgeons will experience losses in hand–eye coordination, dexterity, and/or time efficiency due to the new device [10, 18, 19]. It has been shown that—generally—surgeons with a long lasting experience in using a conventional microscope harbor drawbacks in the use of a gesture controlled, robotic exoscope as compared to younger surgeons with little or no microscope history [17]. Further, recent comparisons of conventional microscopes with monitor-based exoscopes showed no significant difference in surgical time [6]. Our participants used the RS from the start of the laminotomy to the finalized decompression with a median duration of 14.8 min. This amount of time is comparable to the reported time for the same procedure using a conventional microscope from the literature, showing that the majority of participants was able to work confidently with the RS quickly after the standardized training [20]. Using the pre-interventional customized ten-step microsurgical course, the authors were able to depict an efficient learning curve with the basic RS commands [17].

Sufficient training plays a key role in user satisfaction, especially in the case of surgical instruments [5, 21]. The application of the RS in thoracolumbar decompression showed an overall user satisfaction of 84%. In particular, high satisfaction was shown for image quality and depth perception, which depended mainly on the setup for each surgeon. The safety of the application of such new devices is especially important for surgical accesses, since in case of complications (e.g., bleeding, injuries of the dura), time-efficient action is required [12, 22]. The satisfaction of the participants after a short training period showed that the RS could be used routinely for thoracolumbar decompression. However, not all participants could terminate the intervention due to symptoms like headache, dizziness, and nausea. Therefore, might the RS need further improvements, especially on the HMD, before it can sufficiently be used by a broad range of surgeons. Nevertheless, it should be mentioned that this analysis only investigated the first intervention of each surgeon with the device. With more frequent use, a more routine handling of the RS can be assumed.

In this study, the authors observed the highest command error rate in the first quarter of surgical time, which can be explained by a lack of experience with the RS in an operative setting. However, the second highest rate was observed for the last quarter, which is not consistent with the authors’ assumption of a learning curve during surgery. The authors attribute this to a detailed high magnification inspection of all areas of the surgical field at the end of decompression, where again many commands are given that were explained but rarely needed before (e.g., automatic HMD-lift up), leading to an increase in the error rate. In the first three quarters, the error rate decreased for similar commands, again indicating an efficient learning curve and rapid adaptation of participants to the RS.

## Limitations

One of the major weaknesses of this study is the lack of a control group, which would have allowed an even better assessment of the differences between the exoscope and the conventional microscope; however, we acknowledge that during the use of a conventional operating microscope for thoracolumbar decompression, no technical assistance would have been necessary and discomfort by posture would have been in the range of previous publications. The use of participants with varying expertise hampered a uniform analysis of the learning curve; furthermore, the number of participants is small. In sum, there is a need for a further clinical investigation including direct comparison of the conventional microscope with the exoscope.

## Conclusion

The results of this cadaver-based study demonstrated rapid adaption of participants to the HMD-based exoscope using the ten-step microsurgical course. With the aid of the RS, sufficient thoracolumbar decompression was achieved that was comparable to the use of a common operating microscope. The post-interventional questionnaires showed overall satisfaction as well as a sufficient image quality and ease of use.
